# Vitrification in Open and Closed Carriers at Different Cell Stages: Assessment of Embryo Survival, Development, DNA Integrity and Stability during Vapor Phase Storage for Transport

**DOI:** 10.1186/1472-6750-11-29

**Published:** 2011-03-30

**Authors:** Faten AbdelHafez, Jing Xu, Jeffrey Goldberg, Nina Desai

**Affiliations:** 1 Cleveland Clinic Fertility Center, Department of OB/GYN and Women's Health Institute, Cleveland Clinic Foundation, USA

**Keywords:** vapor phase nitrogen, liquid nitrogen, contamination, IVF, dry shipper, transportation, vitrification, closed carrier, cryoloop, Cryotip, HSV straw, storage, embryo

## Abstract

**Background:**

High cooling rates with vitrification can be achieved through the use of carriers that allow cryopreservation in fluid volumes < one μl. Open carriers allow direct contact of embryos with liquid nitrogen (LN2) whereas closed carrier systems sequester the embryo within a sealed system during immersion in LN2. The use of closed systems may be preferable to reduce the possibility of cross-contamination. In the present study, we compare open and closed carriers for vitrification of embryos. We also examine their ability to retain embryo viability during vapor phase transport.

**Methods:**

Frozen one-cell mouse embryos were thawed and randomly allocated to treatment groups. Embryos were cultured and vitrified at the 8-cell (CL) or at the blastocyst (BL) stage. The cryoloop, an open carrier was tested against two closed systems, the Cryotip and the HSV straw. Carriers were tested for their ability to maintain embryo viability when held in the vapor phase of a dry shipper for a period of 96 hours. Outcome parameters monitored were embryo survival, recovery, subsequent development and signs of DNA damage.

**Results:**

A total of 561 embryos were vitrified. The only parameter significantly affected by the type of carrier was the percentage of embryos recovered after warming. Vitrification of both CL and BL stage embryos in the Cryotip resulted in significantly lower recovery rates (P < 0.001). The subsequent developmental parameters were unaffected by either the carrier or the cell stage. Vapor phase storage for 96 hours under "transport conditions" did not appear to adversely affect the viability after warming. Quantitative analysis for DNA damage showed that <5% of cells were TUNEL positive. Interestingly, the overall percent of cells exhibiting DNA damage was lower after CL stage vitrification (P < 0.001).

**Conclusion:**

This study is one of the first to examine DNA integrity after vitrification on different carriers and at different cell stages. It also provides insight on relative safety of short term vapor storage of vitrified embryos during transport. Within the limits of this study we could not detect an adverse effect of vapor storage on blastomere DNA or other measured outcome parameters.

## Background

Vitrification for human embryo cryopreservation is a promising technique but requires the use of carriers that allow ultra-rapid reduction in temperature [1]. To this end, the volume of fluid during cryopreservation is often reduced to less than 1 μl through the use of specially designed vitrification carriers. One such carrier, the cryoloop, allows direct contact of the embryos with liquid nitrogen (LN2), resulting in cooling rates of over 20,000°C/min [2]. Moreover, the cryoloop stays immersed in the LN2 within the cryovial itself. Theoretical concerns regarding contamination during direct exposure and storage of cryopreserved samples in LN2 have been raised [3]. The source of microbial and/or viral contamination could be from the LN2 source itself or cross-contamination from an infected sample in the storage dewar.

Although no infections have been demonstrated with oocyte/embryo storage to date, there is a movement towards the use of closed vitrification carriers. Several closed carrier systems, some FDA approved for specific cell stages, are now commercially available: the Cryotip (Irvine Scientific, CA, USA), high security vitrification (HSV) straw (Cryo BioSystem, Paris, France), VitriSafe (VitriMed, Austria), the Cryopette (Origio, Denmark) and the Rapid-i ™(Vitrolife Sweden AB). Other vitrification approaches include the S3 system [4] and the S3 μS-VTF device [5], which adapt existing FDA approved products for use during vitrification. Several recent publications have examined commercially available closed carriers for human embryo vitrification [6-10].

One concern with closed vitrification carriers is whether the rate of cooling is sufficient to minimize cell damage in both cleavage and blastocyst stage embryos. Another consideration is the ability of vitrified embryos to sustain viability when held only in the vapor phase for 3-4 days during transportation. Dry shippers for transporting embryos are designed to keep the sample at -150°C in the vapor phase of LN2. Vitrified embryos in closed carriers are completely sequestered from the LN2 and are therefore potentially more vulnerable to temperature variations, especially when stored in vapor phase versus directly in the LN2 phase. The effectiveness of different carriers for transporting embryos during vapor phase storage has never been evaluated or compared. Measurement of DNA fragmentation in embryos upon warming may help to further gauge environmental effect on embryo quality [11].

The objective of the current investigation was to compare the performance of two closed vitrification carriers, the HSV straw and the Cryotip, to the open carrier cryoloop. Positive outcomes, including live births, from embryos vitrified with the cryoloop have been well-documented in the literature [12-18]. We performed a detailed assessment of each carrier for both cleavage and blastocyst stage vitrification. The effect of vapor phase storage on embryonic development and DNA integrity in vitrified embryos was also examined.

## Methods

### Study Design

The experiments were carried out in two parts. In experiment 1 we examined the effect of the vitrification carrier on embryo survival, recovery and subsequent development. We also looked for signs of DNA damage within the embryo. We tested embryos vitrified at both the 8-cell cleavage stage (Day 3) and the blastocyst stage (Day 5). In experiment 2, our objective was to examine the effect of vapor phase storage on embryos vitrified in open and closed carriers. To this end the vitrified embryos were held in the vapor phase of a dry shipper for a period of 96 hours to mimic transport. Once again we assessed embryo survival, developmental characteristics and tested for DNA integrity in the blastomeres.

Commercially available frozen one-cell mouse embryos (Conception Technologies; San Diego, CA, USA) were used for all of the above experiments. Thawed embryos were pooled and cultured at 37°C with 5% CO_2 _in air in a humidified incubator. The following morning 2-cell embryos were randomly allocated to treatment groups and cultured. Embryos were vitrified at the 8-cell stage (CL) or at the blastocyst stage (BL). We tested the cryoloop an open carrier (Hampton Research, Laguna, CA, USA) against two closed vitrification carrier systems, the Cryotip (Irvine Scientific, CA, USA) and the HSV straw (Cryo BioSystem, Paris, France).

### Vitrification of embryos in different carriers

Embryos were exposed to the first vitrification solution (Vit 1) composed of 7.5% dimethylsulphoxide (DMSO) and 7.5% ethylene glycol (EG) in basal medium either for 2 minutes, for cleavage stage embryos, or 3 minutes for blastocysts. The additional time given for blastocysts was based on our clinical experience with blastocyst vitrification ([19] and unpublished data). Embryos were then moved to a second vitrification solution (Vit 2) composed of 15% DMSO/EG, 10 μg/ml Ficoll and 0.65 mol/L sucrose for 45 seconds. The basal medium for cryoprotectant (CPA) preparation was Global Blastocyst medium (Life Global, Ontario, Canada) supplemented with 20% Synthetic Serum Substitute (SSS; Irvine Scientific, USA). All the steps were performed at 37°C. After the final exposure to CPA, the embryos were quickly loaded on to the assigned vitrification carrier.

#### Cryoloop

A thin film of CPA was applied to the cryoloop by first dipping the loop in Vit 2. Embryos were then picked up with a finely drawn micropipette and quickly deposited on the cryoloop. The cryoloop with embryos was immediately immersed in a cryovial pre-filled with LN2.

#### HSV straw

Embryos were loaded on to the cut-off end of the microcapillary tube. The embryos were placed in the gutter close to the open end of the tube in a miniscule amount of fluid (<0.5 μl). The capillary tube was then inserted in to a straw. The open end of the straw was then closed using a heat sealer (Cryo BioSystem, Paris, France). The straw with embryos was then plunged in LN2.

#### Cryotip

Embryos were loaded in the Cryotip carrier as directed by the vendor. Briefly, with the aid of a connector and a Hamilton syringe, a small volume of Vit 2 is aspirated followed by the embryos in Vit 2 then another small volume of Vit 2. The lower end was carefully heat sealed and the cover sleeve was slid down. The connector and syringe were then carefully removed followed by heat sealing the thicker upper end of the Cryotip. Finally, the covered Cryotip was plunged directly into LN2 for vitrification.

### Warming and recovery of embryos

Embryos were unloaded from the carrier into warming solution 1 (WS1) containing 0.25 M sucrose in Global Blastocyst medium with 20% SSS. After 2 minutes they were pipetted into warming solution 2 (WS2) containing 0.125 M sucrose for 3 minutes. All warming steps were performed at 37°C. Embryos were unloaded from the different carriers as follows: (1) Cryoloop: embryos were recovered by quickly immersing the loop in WS1. Embryos displaced from the cryoloop were visualized with a dissecting scope. (2) HSV: the straw was held in LN2, while the upper end of the outer sheath was cut using wire cutters. The inner straw with gutter was quickly pulled out of the sheath using the handling rod and immersed in WS1 to unload the embryos. (3) Cryotip: the tip was initially warmed by immersion in a 37°C water bath for 3 seconds. After drying the outside of the Cryotip, the sealed end at the top of the tip was cut off using scissors. A Hamilton syringe was affixed to the end of the Cryotip using a silicon connector. The metal cover was pushed upwards to expose the fine Cryotip containing the embryos. The sealed tip was cut and embryos were quickly expelled in to WS1.

Embryos were cultured in Global Medium with 10%SSS at 37°C with 5.5% CO2 in air. Embryos vitrified at the cleavage stage were allowed to grow for 48 hours after warming. Their progression to the blastocyst stage was monitored before termination of the experiment. Vitrified-warmed blastocysts were kept in culture for 3 hours to assess re-expansion.

### Storage of vitrified embryos

In experiment 1, embryos vitrified on the three different carriers were completely immersed in LN2 and stored in the liquid phase. In experiment 2, we wanted to compare the effectiveness of the three different carriers for vapor phase storage as would be necessary during transport of vitrified embryos. The embryos were vitrified as per the standard protocol using the different carriers then immersed in LN2. For each carrier, one half of the vitrified embryos were randomly allocated to be held in the vapor phase for 96 hours, while the other half remained immersed in LN2. We tested embryos vitrified at both the cleavage and blastocyst stage.

### DNA Damage

At the conclusion of the experiments, all vitrified-warmed embryos were assessed for cryo-injury. DNA damage was assessed at 48 hours for embryos vitrified at the cleavage stage and grown in culture. For blastocysts the evaluation was performed 3 hours after warming. The embryos were rinsed in PBS and fixed in 4% paraformaldehyde for 30 minutes at room temperature. After washing, the embryos were permeabilized with 0.1% Triton X-100 on ice for 10 minutes followed by two PBS washes. DNA damage in the embryos was assessed using the In-Situ Cell Death Detection kit - TMR-Red (Boehringer Mannheim, Minneapolis, MN). This kit uses TdT-mediated dUTP nick labeling (TUNEL technique) to detect nuclei with fragmented DNA. The embryos were incubated with the TUNEL reaction mixture for 60 minutes at 37°C in the dark. Negative and positive controls were included for each experiment. The negative controls were non-vitrified embryos. The positive controls were treated with DNAse for 10 minutes to induce DNA strand breaks. Embryos were washed three times, mounted in 4', 6-diamidino-2-phenylindole (DAPI) to label all nuclei. Embryos were examined under a fluorescent microscope (Olympus) and the images were captured for analysis. Total cell number per embryo and the number of cells with DNA damage were tabulated for each treatment, carrier and vitrification cell stage. The percent DNA damage was calculated for each embryo by dividing the number of TUNEL positive cells by the total blastomere count.

### Outcome measures and statistical analysis

Main outcome measures were as follows: (a) Recovery; % of vitrified embryos successfully recovered from the carrier upon warming (b) Survival; % of recovered embryos having greater than half of their cells intact immediately after warming (c) Blastocyst development; % of vitrified-warmed cleavage stage embryos developing to blastocyst after 48 hours of culture (d) Re-expansion; % of vitrified-warmed blastocysts re-expanded 3 hours after warming (e) Total blastomere number; cell count in vitrified-warmed embryos at termination of experiment (Day 5 for both CL and BL vitrification) (f) DNA damage at termination of experiment; % of total cells showing TUNEL labeling.

Statistical analysis was performed using the Stats Direct program (StatsDirect Ltd, UK). The chi square and ANOVA tests were used as appropriate. P values of <0.05 were considered to be significant.

## Results

### Experiment 1

**Figure 1:**
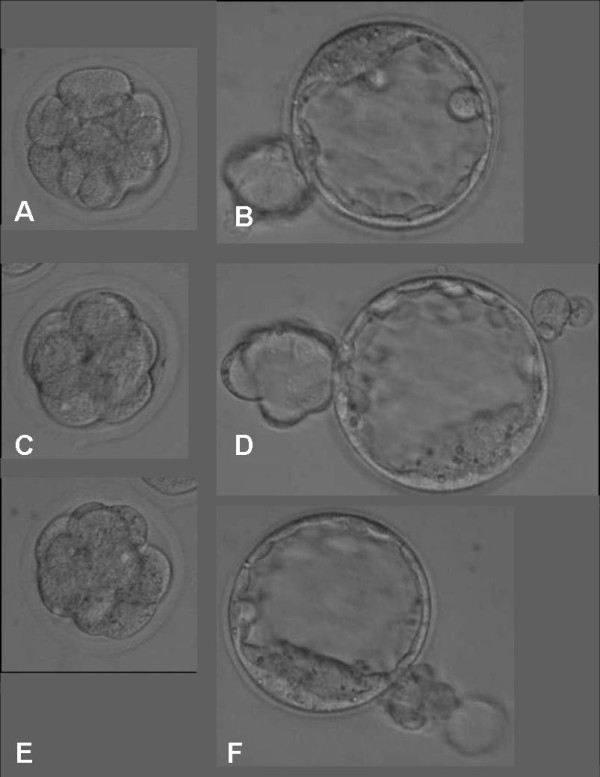
**Cleavage stage embryos vitrified on different carriers and stored in liquid nitrogen**. Images taken immediately after warming (A, C, E) and 48 hours later (B, D, F). A-B: Cryoloop, C-D: HSV straw, E-F: CryoTip.

**Figure 2:**
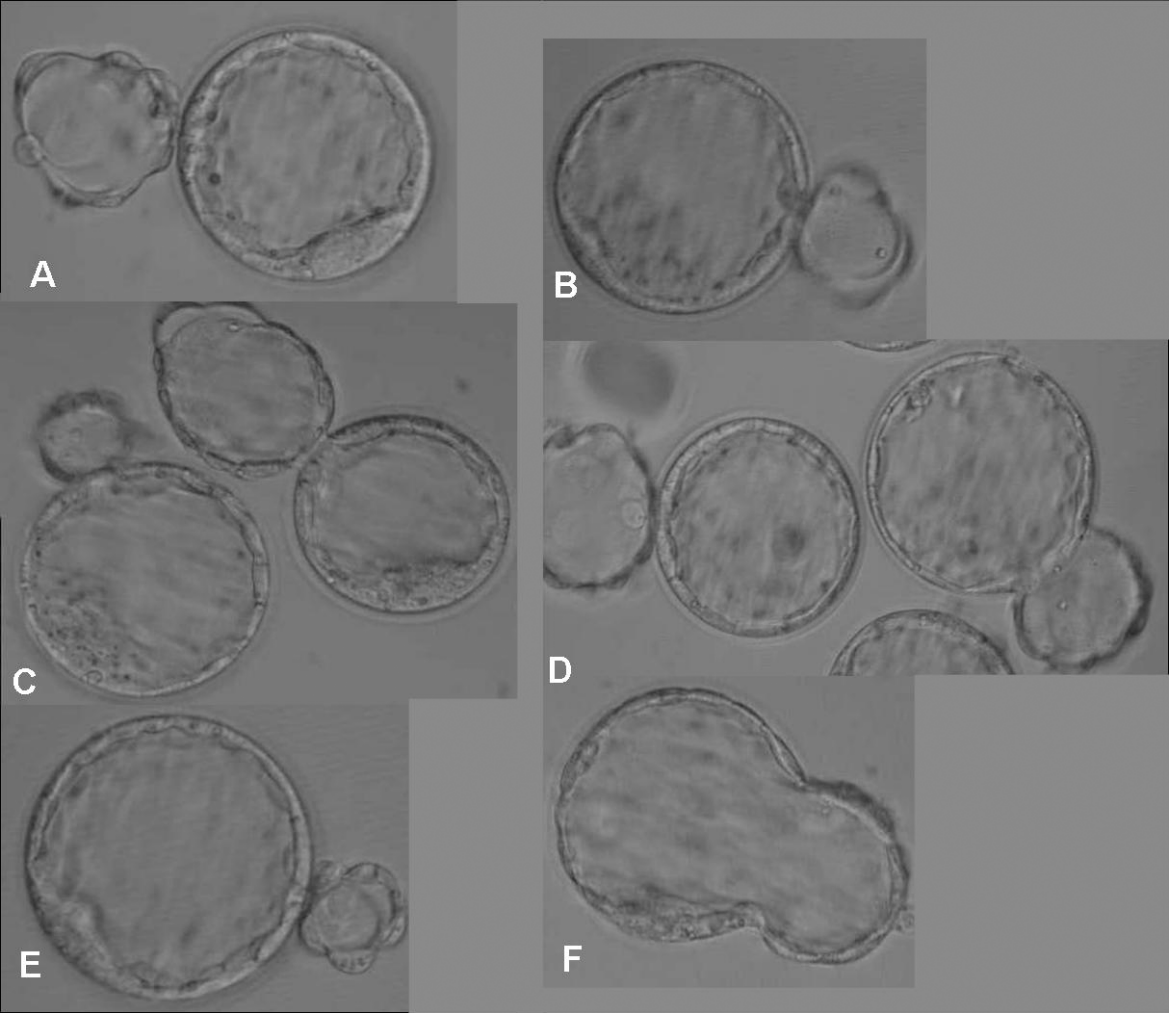
**Blastocysts vitrified on different carriers and stored in liquid nitrogen**. Photographed before vitrification (A, C, E) and three hours after warming (B, D, F). A-B: Cryoloop, C-D: HSV straw, E-F: CryoTip.

To compare the effectiveness of the different carriers for embryo vitrification, 179 cleavage stage embryos (CL) and 151 blastocysts (BL) were vitrified using the cryoloop, the HSV straw or the Cryotip. Figures 1 and 2 depict the morphology of embryos vitrified at cleavage and blastocyst stages with the different carriers. Warmed embryos showed little evidence of cell damage. Table 1 summarizes the results for the different cell stages at vitrification as well as the three different carriers tested. The only parameter that appeared to be significantly affected by the type of carrier was the percentage of embryos recovered after warming. Vitrification of both cleavage and blastocyst stage embryos in the Cryotip resulted in significantly lower recovery rates (P < 0.001). The subsequent developmental parameters i.e. growth to blastocyst (CL vitrified embryos), re-expansion (BL vitrified embryos) and overall blastomere cell count were unaffected by either the carrier or the initial cell stage. The open versus closed vitrification systems did not overtly affect the degree of cryo-injury. The percentage of blastomeres per embryo showing DNA damage was similar between carriers. However, there was a significantly higher rate of DNA damage after vitrification at the blastocyst stage compared to cleavage stage embryos (P < 0.0001).

**Table 1:** Vitrification of cleavage and blastocyst stage embryos using open and closed carriers

Cleavage Stage Vitrification						
**Carrier**	**Total embryos** **(n)**	**Recovery** **(%)**	**Survival** **(%)**	**Blastocyst formation after** ** 48 hour culture (%)**	**Total blastomeres** ** (mean ± SD)**	**% DNA Damage** **(mean ± SD)**

**Cryoloop**	60	100	100	95	81.9 ± 14.0	1.85 ± 2.05

**HSV**	52	100	100	94	82.5 ± 15.6	2.06 ± 1.50

**Cryotip**	67	85*	100	98	78.6 ± 17.9	2.12 ± 2.04

**Blastocyst Stage Vitrification**						

**Carrier**	**Total embryos** **(n)**	**Recovery** **(%)**	**Survival** **(%)**	**Re-expansion** **(%)**	**Total blastomeres** ** (mean ± SD)**	% DNA Damage ** (mean ± SD)

**Cryoloop**	44	100	100	100	86.4 ± 25.8	4.36 ± 2.72

**HSV**	55	100	100	100	85.9 ± 23.7	3.34 ± 2.79

**Cryotip**	52	75 *	79	79	88.0 ± 19.2	3.41 ± 2.66

* Significantly lower recovery than with other carriers. P = 0.0001

** Percent DNA damage was higher in embryos vitrified at the blastocyst versus

cleavage stage (P < 0.0001), regardless of the type of carrier

### Experiment 2

The ability of the different carriers to sustain vitrified embryo potential when held in the vapor phase was tested in this experiment. The LN2 shipper routinely used for transporting embryos was charged overnight with LN2. Vitrified embryos stored in the vapor phase for 96 hours were critically assessed after warming and culture. The data was compared to that observed with the control group stored in LN2. A total of 231 vitrified embryos (CL = 115; BL = 116) were randomly allocated to the different treatment groups. These data are summarized in Table 2. For cleavage stage embryos, liquid and vapor phase storage resulted in comparable survival and blastocyst formation rates. The type of carrier did not influence these outcome parameters. The average blastomere counts were also unaffected by being held in the vapor phase before warming and extended culture to blastocyst. We were also unable to detect an overt negative impact of vapor storage on vitrified blastocysts. Post-warming survival, re-expansion, and total blastomere count were quite similar between the carriers, independent of storage condition.

**Table 2:** Short term vapor storage of vitrified embryos on different carriers to simulate transport conditions

	Cleavage Stage Vitrification					
**Carrier**	**Cryoloop** **(n = 40)**		**HSV Straw** **(n = 35)**		**Cryotip** **(n = 40)**	

**Storage Condition**	**LN2**	**Vapor**	**LN2**	**Vapor**	**LN2**	**Vapor**

**Survival (%)**	100	100	100	100	100	100

**Development to blastocyst after 48 hours (%)**	100	100	100	93	100	100

**Total blastomeres^a ^(mean ± SD)**	82.21 ± 13.28	89.18 ± 18.52	87.20 ± 10.67	88.5 ± 9.55	81.10 ± 14.09	75.53 ± 17.62

						

	**Blastocyst Stage Vitrification**					

**Carrier**	**Cryoloop** **(n = 41)**		**HSV straw** **(n = 40)**		**Cryotip** **(n = 35)**	

**Storage Condition**	**LN2**	**Vapor**	**LN2**	**Vapor**	**LN2**	**Vapor**

**Survival (%)**	100	100	100	100	100	100

**Re-expansion (%)**	90	81	95	85	80	85

**Total blastomeres^a ^(mean ± SD)**	108 ± 18	96 ± 19	96 ± 20	90 ± 20	96 ± 4	86 ± 19

^a ^Total cell count at termination of experiment for both Vit-CL and Vit-BL on day 5

No significant difference in survival, development, re-expansion or cell number after short term vapor storage as compared to liquid nitrogen

**Figure 3:**
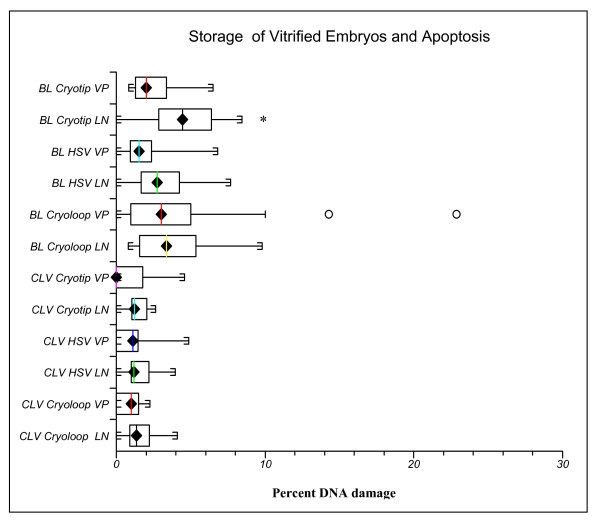
**Cleavage and blastocyst stage embryos were vitrified in different carriers and stored in liquid nitrogen (LN) or held in the vapor phase (VP) of a liquid nitrogen dry shipper for 96 hours to simulate transport conditions**. Upon warming, DNA damage was assessed by quantification of the percentage of blastomeres per embryo exhibiting DNA fragmentation. The only carrier to exhibit a difference was the Cryotip, with a significantly higher percentage of DNA damage with blastocyst storage in the liquid phase. *P = 0.004. The percentage of DNA damage was significantly higher with vitrification of blastocysts versus cleavage stage embryos (P < 0.001).

**Figure 4:**
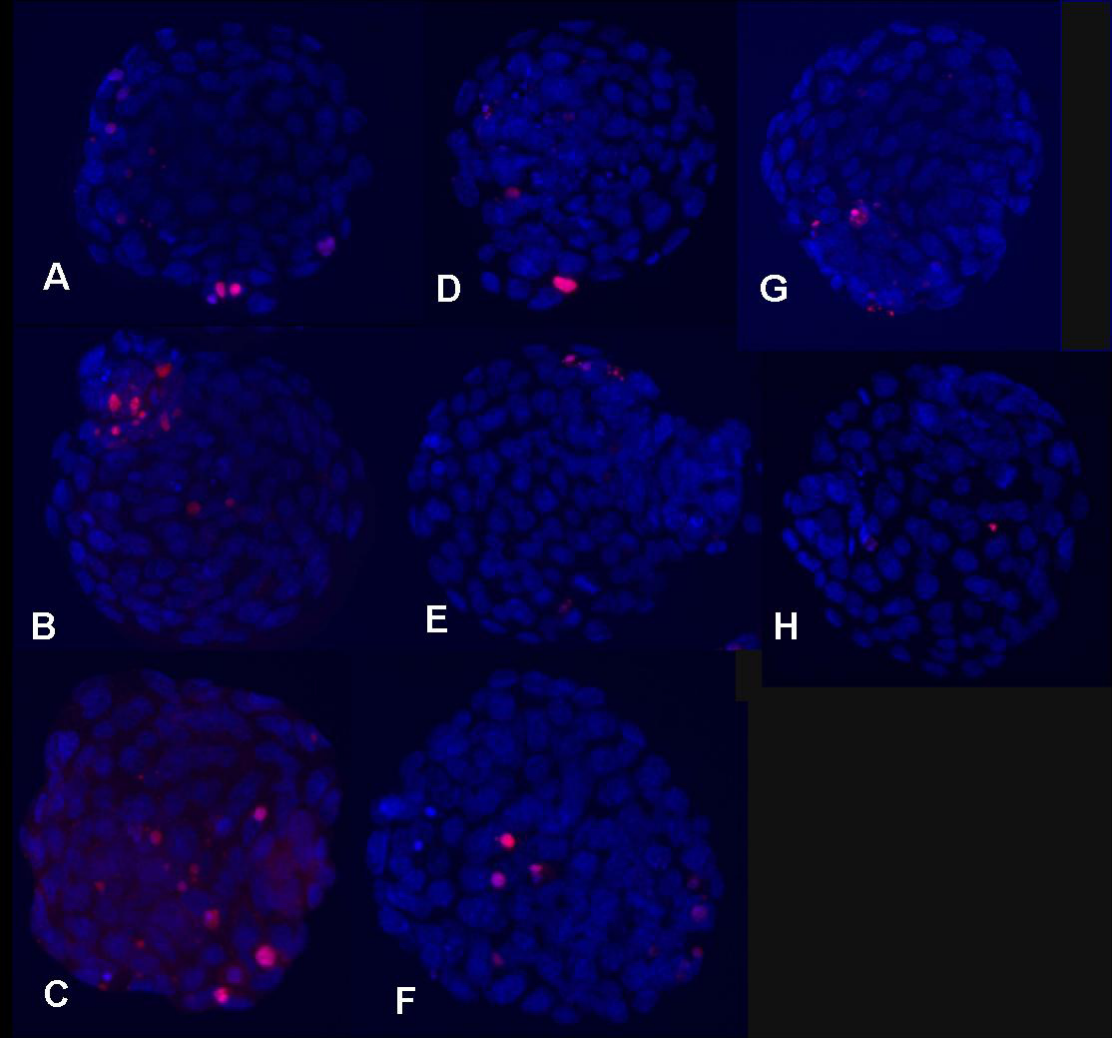
**Fluorescent micrographs with representative images of DNA damage detected in vitrified-warmed mouse blastocysts**. Nuclei of blastomeres stained with DAPI showing blue fluorescence. Nuclei with DNA strand breaks were labeled using the TUNEL assay. Damaged cells bound TMR-red dUTP and exhibited red fluorescence. A-C: Blastocysts vitrified on CryoTip and stored in LN2. TUNEL staining was performed 3 hours after warming. D-E: Blastocysts vitrified on CryoTip and stored in vapor phase under transport conditions. Stained 3 hrs post warming. G-Blastocyst vitrified on cryoloop and stored in LN2 (3 hrs post warming). H-Cleavage stage embryo vitrified on cryoloop and stored in LN2. TUNEL labeling 48 hours after warming.

Figure 3 compares DNA damage after storage for 96 hours in the vapor phase of LN2 to controls immersed in LN2. Interestingly, vitrified blastocysts stored in the liquid phase with the Cryotip showed more DNA damage than their counterparts held in the vapor phase (P = 0.004). Incomplete sealing of the Cryotip may have entrapped LN2 which negatively impacted recovery and blastomere survival during warming. With vapor storage before warming, LN2 within the Cryotip would have had ample time to dissipate. The DNA damage index was higher in blastocyst versus cleavage stage embryos. Figure 4 shows examples of vitrified warmed embryos stained for DNA damage.

## Discussion

The current investigation carefully details embryonic survival and development after vitrification on an open carrier, the cryoloop, and two closed carriers, the HSV straw and the Cryotip. All three carriers were effective for both cleavage and blastocyst vitrification. The Cryotip was a bit more vulnerable to technical difficulties during recovery. Based on the outcome parameters designated in this study, holding vitrified embryos in the vapor phase of LN2 did not result in obvious impairment of development or a significant increase in damage to cellular DNA. This finding is reassuring, suggesting that transport of vitrified embryos may be possible with minimal harm. Clinical outcome data from transported embryos is still needed to further corroborate these data.

The theoretical risk of cross-contamination in LN2 containers even at -196°C has been widely debated [3,20]. Even if source LN2 is sterile, it can become contaminated by various routes including handling or contact with infectious samples. The new European Directive on tissue storage, as well as FDA requirements, for new cryopreservation devices mandate the use of closed systems to negate any risk of microbial or viral contamination. To date there are only a few commercially available closed carriers for vitrification, the Cryotip [6,7], the HSV straw [8], VitriSafe [9], the Cryopette [10] and more recently the Rapid-i™[21]. This latter system is modeled after the cryoloop, where the embryo is placed in a miniscule drop suspended in an open hole on a cylindrical stick, which then can be completely enclosed and sealed, so there is no direct contact with LN2.

One of the concerns with the closed carrier systems is the slower rate of cooling and warming and how this might affect subsequent outcomes. The cooling rate with the cryoloop and other open carriers has been reported to be close to -20,000°C/min [6,15,22]. The cooling rate with the Rapid-i™device has been measured at -1220°C/min and the warming rate was six-fold higher [21]. Kuwayama et al (2005) reported that the cooling rates with the open carrier Cryotop and the closed Cryotip carrier were -23,000°C/min and -12,000°C/min respectively [6]. Both carriers yielded comparable post warming blastocyst survival, pregnancy and delivery rates. Cooling rates for Vitrisafe and the HSV straw are very similar, in the range of -1300 °C [9]. The authors suggest that perhaps any decrease in the cooling rate should be compensated for by a gradual increase in the intracellular cryoprotectant concentrations.

In the current investigation, no modifications were made to the vitrification protocol to accommodate closed versus open carriers. There was no evidence of poorer survival or developmental parameters as a result of the slower cooling rates in the closed carriers. DNA damage in blastomeres was comparable in embryos vitrified on the open cryoloop and the closed HSV straw. The Cryotip was more subject to problems during embryo recovery. A study comparing the Cryotip to the Cryopette [10] also noted a lower recovery rate, (81% versus 100%, respectively).

It has been suggested that the warming rate may actually play a more dominant role in modulating survival rates after vitrification than the cooling rate [23]. These investigators subjected oocytes to different cooling and warming rates during the vitrification procedure. Regardless of the cooling rate, oocyte survival could be reduced to 0% if the warming rate was at the lowest level. In contrast, survival was over 80% if the highest warming rate (+1827)°C/min was combined with any cooling rate. The authors conclude that too slow warming is lethal to cells, allowing tiny ice crystal formation. This may in part explain why closed carrier systems with their slower cooling rates can still be successfully applied, as long as the design parameters allow for sufficiently fast warming rates. The few published studies comparing pregnancy outcomes using open versus closed vitrification systems suggest that both can be equally effective [6,9].

Cryopreservation induced stress results in an increase in apoptotic gene expression, DNA fragmentation and reduces developmental capacity [11]. In the current study, DNA damage in vitrified-warmed blastocysts ranged from an average of 1.9% to 4.7%. In contrast, embryos vitrified at the 8-cell stage and subsequently warmed and cultured to blastocyst were observed to have a lower percentage of blastomeres with damaged DNA, less than 2% (P < 0.0001). With cleavage stage vitrification, the damaged cell(s) represent a very small proportion of the embryo by the time it undergoes several cleavages and reaches the blastocyst stage two days later. We attribute the wider range of DNA damage during blastocyst vitrification to differences in blastocoel shrinkage after exposure to vitrification solutions (VS). Despite the fact that all blastocysts were fully expanded at the time of vitrification, their response to VS solutions varied. Some collapsed immediately while others still had some blastocoelic fluid at the end of the incubation period before loading on to the carriers. Mechanical blastocoelic fluid reduction prior to vitrification has been suggested by several studies as an effective technique tool for maximizing blastocyst post-warming survival[14,24-27] and reducing DNA damage [27].

Short term vapor storage for transport as well as long term storage of cryopreserved samples in the LN2 vapor phase has been an area of much interest. Vapor storage has been applied to cryopreserved human sperm [28,29], mouse embryos vitrified on EM grids [30] and human oocytes vitrified on the Cryotop [31]. In the latter study, live birth rates were shown to be unaffected by storage method. All of the studies concluded that vapor phase storage was as efficient as liquid phase storage. Vapor phase storage has been proposed as a means to circumvent the risk of sample cross-contamination, especially where open vitrification carriers are stored directly in LN2.

However, a recent study suggests that storage in the vapor phase still presents an inherent risk for pathogen transmission and cross-contamination [32]. Ultimately, only a closed, sealed cryopreservation system can ensure risk-free storage of samples. Reproductive laboratories will need to transition to such systems.

## Conclusions

In conclusion, this study is one of the first to examine DNA integrity after vitrification on different carriers and at different cell stages. It also provides insight on the relative safety of short term vapor storage of vitrified embryos during transport. Within the limits of this study we could not detect an adverse effect of vapor storage on blastomere DNA or other measured outcome parameters. A further limitation of this work was the use of frozen mouse embryos. Monitoring of outcomes with vitrified human embryos that have been transported between clinics is imperative to fully appreciate the risks associated with vapor phase storage. The current data set adds to the pool of knowledge on closed vitrification systems and their efficacy in allowing cooling at sufficient rates to successfully vitrify embryos at both early and late stages.

## Authors' contributions

FA: contributed in writing and data analysis, ND: contributed in data analysis and writing, JX: performed the experiments and helped tabulate data, and JG: critically reviewed the manuscript. All authors read and approved the final manuscript.

## References

[B1] Rall WF Fahy GM Ice-free cryopreservation of mouse embryos at -196 degrees C by vitrification Nature 1985 313 573 575 10.1038/313573a0 3969158

[B2] Shaw JM Jones GM Terminology associated with vitrification and other cryopreservation procedures for oocytes and embryos Hum Reprod Update 2003 9 583 605 10.1093/humupd/dmg041 14714593

[B3] Bielanski A Vajta G Risk of contamination of germplasm during cryopreservation and cryobanking in IVF units Hum Reprod 2009 24 2457 2467 10.1093/humrep/dep117 19561041

[B4] Stachecki J S3 Vitrification System: a novel approach to blastocyst freezing Fertility magazine 2009 11 30 36

[B5] Schwiewe M MicroSecure Vitrification (uS-VTF) Procedures:Optimum simplicity, security, cost-savings and effectiveness combining FDA-approved products Journal of Clinical Embryology 2010 13 33 40

[B6] Kuwayama M Vajta G Ieda S Kato O Comparison of open and closed methods for vitrification of human embryos and the elimination of potential contamination Reprod Biomed Online 2005 11 608 614 10.1016/S1472-6483(10)61169-8 16409712

[B7] Cobo A Perez S De los Santos MJ Zulategui J Domingo J Remohi J Effect of different cryopreservation protocols on the metaphase II spindle in human oocytes Reprod Biomed Online 2008 17 350 359 10.1016/S1472-6483(10)60218-0 18765005

[B8] Camus A Clairaz P Ersham A Van Kappel AL Savic G Staub C [The comparison of the process of five different vitrification devices] Gynecol Obstet Fertil 2006 34 737 745 10.1016/j.gyobfe.2006.07.017 16962814

[B9] Vanderzwalmen P Ectors F Grobet L Prapas Y Panagiotidis Y Vanderzwalmen S Stecher A Frias P Liebermann J Zech NH Aseptic vitrification of blastocysts from infertile patients, egg donors and after IVM Reprod Biomed Online 2009 19 700 707 10.1016/j.rbmo.2009.09.011 20021718

[B10] Portman M Nagy ZP Behr B Evaluation of blastocyst survival following vitrification/warming using two different closed carrier systems Human Reproduction 2010 25 Suppl 1 P 375

[B11] Park SY Kim EY Cui XS Tae JC Lee WD Kim NH Park SP Lim JH Increase in DNA fragmentation and apoptosis-related gene expression in frozen-thawed bovine blastocysts Zygote 2006 14 125 131 10.1017/S0967199406003649 16719948

[B12] Desai N Abdelhafez F Bedaiwy M Goldberg J Falcone T Goldfarb J Clinical Pregnancy and Live Births After Transfer of Embryos Vitrified On Day 3 Reprod Biomed Online 2010 in press 10.1016/j.rbmo.2010.02.010 20378417

[B13] Desai N Blackmon H Szeptycki J Goldfarb J Cryoloop vitrification of human day 3 cleavage-stage embryos: post-vitrification development, pregnancy outcomes and live births Reprod Biomed Online 2007 14 208 213 10.1016/S1472-6483(10)60789-4 17298725

[B14] Mukaida T Oka C Goto T Takahashi K Artificial shrinkage of blastocoeles using either a micro-needle or a laser pulse prior to the cooling steps of vitrification improves survival rate and pregnancy outcome of vitrified human blastocysts Hum Reprod 2006 21 3246 3252 10.1093/humrep/del285 16936299

[B15] Mukaida T Takahashi K Kasai M Blastocyst cryopreservation: ultrarapid vitrification using cryoloop technique Reprod Biomed Online 2003 6 221 225 10.1016/S1472-6483(10)61713-0 12676003

[B16] Takahashi K Mukaida T Goto T Oka C Perinatal outcome of blastocyst transfer with vitrification using cryoloop: a 4-year follow-up study Fertil Steril 2005 84 88 92 10.1016/j.fertnstert.2004.12.051 16009162

[B17] Rama Raju GA Haranath GB Krishna KM Prakash GJ Madan K Vitrification of human 8-cell embryos, a modified protocol for better pregnancy rates Reprod Biomed Online 2005 11 434 437 10.1016/S1472-6483(10)61135-2 16274602

[B18] Balaban B Urman B Ata B Isiklar A Larman MG Hamilton R Gardner DK A randomized controlled study of human Day 3 embryo cryopreservation by slow freezing or vitrification: vitrification is associated with higher survival, metabolism and blastocyst formation Hum Reprod 2008 23 1976 1982 10.1093/humrep/den222 18544577

[B19] Desai N Falcone T Goldberg E Austin C Goldfarb J What is the optimal stage for embryo vitrification-a comparison of embryo survival and clinical outcomes with day 3 cleavage versus blastocyst stage vitrification Fertility and Sterilty 2010 94 S110 10.1016/j.fertnstert.2010.07.454

[B20] Bielanski A Nadin-Davis S Sapp T Lutze-Wallace C Viral contamination of embryos cryopreserved in liquid nitrogen Cryobiology 2000 40 110 116 10.1006/cryo.1999.2227 10788310

[B21] Tarakanov Y Johansson B Lehmann H Appell SP Numerical Simulations Demonstrate Safe Vitrification and Warming of Embryos Using the Rapid-i Device Proceedings of the COMSOL Conference; Milan, Italy 2009

[B22] Lane M Schoolcraft WB Gardner DK Vitrification of mouse and human blastocysts using a novel cryoloop container-less technique Fertil Steril 1999 72 1073 1078 10.1016/S0015-0282(99)00418-5 10593384

[B23] Seki S Mazur P The dominance of warming rate over cooling rate in the survival of mouse oocytes subjected to a vitrification procedure Cryobiology 2009 59 75 82 10.1016/j.cryobiol.2009.04.012 19427303 PMC2729265

[B24] Vanderzwalmen P Bertin G Debauche C Standaert V van Roosendaal E Vandervorst M Bollen N Zech H Mukaida T Takahashi K Schoysman R Births after vitrification at morula and blastocyst stages: effect of artificial reduction of the blastocoelic cavity before vitrification Hum Reprod 2002 17 744 751 10.1093/humrep/17.3.744 11870130

[B25] Chen SU Lee TH Lien YR Tsai YY Chang LJ Yang YS Microsuction of blastocoelic fluid before vitrification increased survival and pregnancy of mouse expanded blastocysts, but pretreatment with the cytoskeletal stabilizer did not increase blastocyst survival Fertil Steril 2005 84 Suppl 2 1156 1162 10.1016/j.fertnstert.2005.03.074 16210007

[B26] Hiraoka K Hiraoka K Kinutani M Kinutani K Blastocoele collapse by micropipetting prior to vitrification gives excellent survival and pregnancy outcomes for human day 5 and 6 expanded blastocysts Hum Reprod 2004 19 2884 2888 10.1093/humrep/deh504 15347597

[B27] Desai N Szeptycki J Scott M AbdelHafez F Goldfarb J Artificial Collapse of Blastocysts Before Vitrification: Mechanical vs. Laser Technique and Effect on Survival, Cell Number, and Cell Death in Early and Expanded Blastocysts Biopreservation and Biobanking 2008 6 3 181 190

[B28] Lim JJ Shin TE Song SH Bak CW Yoon TK Lee DR Effect of liquid nitrogen vapor storage on the motility, viability, morphology, deoxyribonucleic acid integrity, and mitochondrial potential of frozen-thawed human spermatozoa Fertil Steril 2010 20381034 10.1016/j.fertnstert.2010.02.051

[B29] Punyatanasakchai P Sophonsritsuk A Weerakiet S Wansumrit S Chompurat D Comparison of cryopreserved human sperm in vapor and liquid phases of liquid nitrogen: effect on motility parameters, morphology, and sperm function Fertil Steril 2008 90 1978 1982 10.1016/j.fertnstert.2007.09.066 18222437

[B30] Eum JH Park JK Lee WS Cha KR Yoon TK Lee DR Long-term liquid nitrogen vapor storage of mouse embryos cryopreserved using vitrification or slow cooling Fertil Steril 2009 91 1928 1932 10.1016/j.fertnstert.2008.02.126 18440528

[B31] Cobo A Romero JL Perez S de Los Santos MJ Meseguer M Remohi J Storage of human oocytes in the vapor phase of nitrogen Fertil Steril 2010 10.1016/j.fertnstert.2009.10.042 20138272

[B32] Grout BW Morris GJ Contaminated liquid nitrogen vapour as a risk factor in pathogen transfer Theriogenology 2009 71 1079 1082 10.1016/j.theriogenology.2008.12.011 19215973

